# Resurrection and characterization of ancestral xylose transporters enhance the capability of xylose uptake in the mixed sugar co-fermentation of Recombinant *Saccharomyces cerevisiae*

**DOI:** 10.1186/s40643-025-00995-1

**Published:** 2026-01-05

**Authors:** Peining Zhang, Zhaoqing He, Huanan Li, Zhengbing Jiang

**Affiliations:** 1 State Key Laboratory of Biocatalysis and Enzyme Engineering, School of Life Sciences, Hubei University, Wuhan, 430062 People’s Republic of China; 2 Hubei Key Laboratory of Industrial Biotechnology, School of Life Science, Hubei University, Wuhan, 430062 People’s Republic of China

**Keywords:** Ancestral sequence reconstruction, Xylose transporter, Glucose/xylose co-utilization, Saccharomyces cerevisiae

## Abstract

Ancestral sequence reconstruction (ASR) offers a revolutionary approach to resurrect functional proteins, yet its potential in transporter engineering remains underexplored. Here, we pioneered the application of ASR to reconstructing ancestral xylose transporters, addressing the persistent challenge of glucose-mediated inhibition of xylose uptake in *Saccharomyces cerevisiae* during xylose co-fermentation. Through rigorous ASR analysis, we reconstructed ancestral xylose transporters (Xt) and selected two candidates—Xt3 (approximately 140 million years old) and Xt7 (approximately 40 million years old)—based on their phylogenetic positioning, degree of sequence divergence from extant homologs, and predicted structural integrity. Functional characterization demonstrated that both Xt3 and Xt7 significantly enhance xylose uptake efficiency and mitigate glucose-induced repression. In fermentation experiments with mixed sugars (40 g/L xylose and 40 g/L glucose) within 72 h, recombinant *S. cerevisiae* expressing Xt3 achieved 22.75 g/L xylose consumption, surpassing the benchmark N326F^Xltr1p^ (16.22 g/L) by 40.27% and outperforming Xt7 (21.36 g/L) by 6.51%, highlighting Xt3 as the most efficient transporter. Molecular docking suggested a potentially more favorable binding mode for xylose in the ancestral transporters (binding affinity: −3.68 kcal/mol for Xt3 vs. −3.15 kcal/mol for N326F^Xltr1p^). Molecular dynamics simulations further demonstrated that the ancestral transporters formed complexes with xylose that exhibited faster convergence to a stable state and maintained significantly greater conformational stability throughout the simulation compared to the N326F^Xltr1p^ complex. These computational insights provide a plausible structural basis for their enhanced performance. This work contributes to the advancement of lignocellulosic biorefinery technology and provides a practical reference for resurrecting other valuable proteins using ASR’.

**Graphical abstract:**

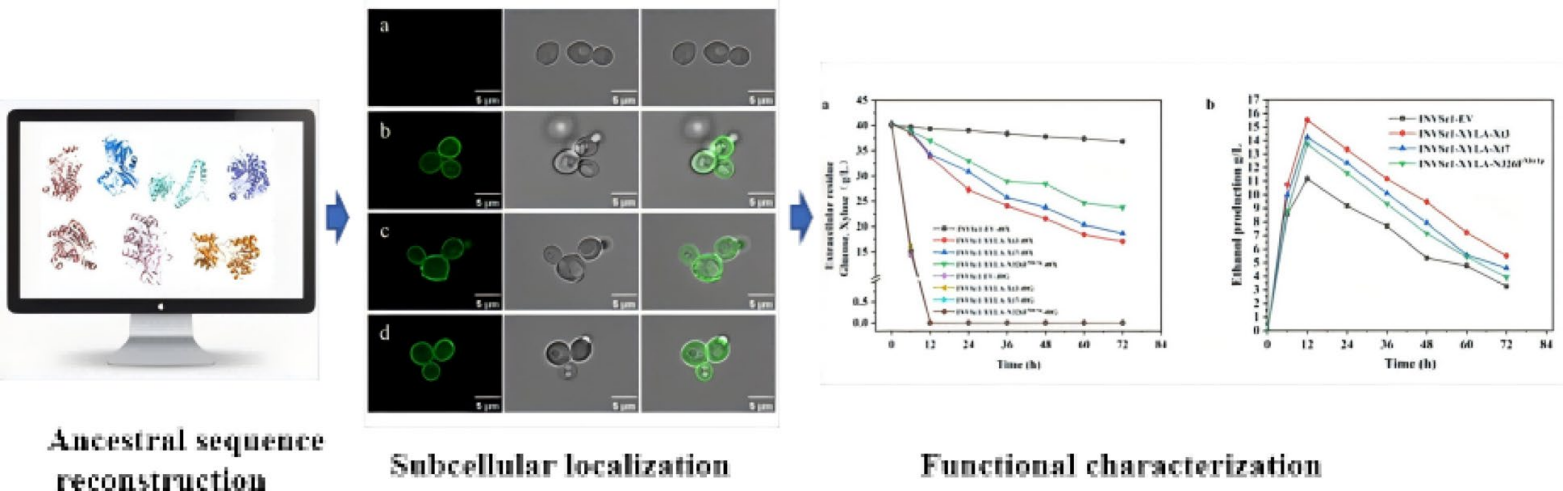

**Supplementary Information:**

The online version contains supplementary material available at 10.1186/s40643-025-00995-1.

## Introduction

 Utilizing renewable lignocellulosic biomass as a replacement for fossil raw materials in the production of bioenergy and chemicals offers a promising approach to mitigate energy crises, reduce greenhouse gas emissions, and decrease environmental pollution (Singh et al. 2022). Lignocellulosic biomass primarily consists of cellulose, hemicellulose, and lignin. While cellulose and hemicellulose are polysaccharides that can be hydrolyzed into fermentable sugars, glucose and xylose emerge as the most abundant monosaccharides in lignocellulosic hydrolysates, constituting approximately 60–70% and 30–40% of the total sugars, respectively (Ragauskas et al. 2014). However, a significant challenge arises during microbial fermentation of these hydrolysates: the efficient utilization of xylose is often inhibited by the presence of glucose (Ning et al. 2021). This glucose-induced repression of xylose metabolism directly limits the overall efficiency of the bioconversion process. Therefore, developing strategies for the effective co-utilization of glucose and xylose presents substantial potential for enhancing the efficiency and economic viability of converting lignocellulosic biomass into bioenergy and valuable chemicals (Nieves et al. 2015).

*Saccharomyces cerevisiae* INVSc1 is widely adopted as a microbial platform for biofuel and biochemical production due to its safety, robustness, and stability (Oh and Jin 2020). However, wild-type *S. cerevisiae* INVSc1 lacks the ability to metabolize xylose. The first successful recombinant *S. cerevisiae* carrying the XI (encoded by xylose isomerase XylA) pathway was achieved by expressing eukaryotic XI from the anaerobic fungus *Piromyces sp* E2 (de Kuyper et al. 2003). An industrial yeast strain carrying integrated CpXylA was evolved in xylose medium, which resulting strain carried CpXylA along with an autonomous replicating sequence element that eventually amplified gene-encoding XI, leading to a high-capacity xylose fermentation strain (Demeke et al. 2015). The strategy of combining metabolic engineering and evolutionary engineering to improve metabolic flux for xylose utilization has been employed to develop engineered *S. cerevisiae* (Su et al. 2020). Most of them utilize the endogenous hexose transporters for xylose uptake, as *S. cerevisiae* lacks a dedicated xylose transport system (Lee et al. 2002). These engineered strains have demonstrated enhanced ability in utilizing xylose when it is used xylose alone as a carbon source; however, during co-fermentation with glucose and xylose, glucose inhibits the transport of xylose in engineered *S. cerevisiae*, due to the low affinity of nonspecific transporters (Hou et al. 2017). This inhibition significantly constrains the co-utilization efficiency of lignocellulosic hydrolysates.

*S. cerevisiae* relies on endogenous glucose transporters (e.g., Hxt1p, Hxt2p, Hxt4p, Hxt5p, Hxt7p, Hxt11p, Gal2p) for xylose uptake (Hamacher et al. 2002). Among these, Hxt7p and Gal2p exhibit the highest xylose transport rates (Wang et al. 2013). However, these transporters display significantly higher affinity for glucose than xylose. Consequently, glucose competitively inhibits xylose uptake during mixed-sugar fermentation. To overcome this limitation, > 80 heterologous transporters from xylose-utilizing microorganisms have been expressed in *S. cerevisiae* (Taveira et al. 2024; Leandro et al. 2006; de Bueno et al. 2020). For example: Gxf1 (*Candida intermedia*) increased xylose utilization rates ~ three-fold (Runquist et al. 2009), Sut1 (*Scheffersomyces stipitis*) enhanced xylose consumption by 25% (Kogje and Ghosalkar 2016), XltR1p (*Trichoderma reesei*) outperformed native Gal2p in xylose transport (Jiang et al. 2020). Despite these advances, most heterologous transporters still prefer glucose over xylose in co-fermentation. To mitigate glucose inhibition, protein engineering strategies (directed evolution, site-directed mutagenesis) have been employed. Through directed evolution of the sugar transporter CiGXS1 FIM, glucose inhibition of xylose transport was eliminated, enabling co-transport of glucose and xylose (Li et al. 2016). The introduction of a variant of the galactose permease Gal2 (Gal2^N376Y/M435I^) significantly reduced glucose repression and enhanced xylose affinity (Rojas et al. 2021). Jiang et al. used homology modeling and molecular docking to predict the XltR1p structure, identifying the N326F mutation that created a xylose-specific transporter (Jiang et al. 2020). Modeling studies, such as motif-based recognition or classification models, have aided in rewiring the sugar transporter preferences in yeast (Young et al. 2014). These advances expand the library of xylose transporters, providing fundamental tools for developing high-performance industrial yeast strains. However, developing novel transporters that simultaneously achieve high xylose affinity, specificity, and insensitivity to glucose inhibition remains a critical unmet need. 

Ancestral sequence resurrection (ASR) computationally reconstructs extinct protein sequences by analyzing phylogenetic relationships among extant homologs (Mascotti 2022). Critically, ASR explores functional sequence spaces probabilistically, generating novel variants distinct from modern proteins while maintaining a high likelihood of functionality—provided accurate sequence alignments are used (Risso et al. 2018). Consequently, ancestral proteins often exhibit unique functional enhancements, including elevated thermostability, catalytic activity, and broad substrate specificity (Gomez-Fernandez et al. 2020). This approach contrasts with conventional engineering methods by accessing evolutionarily validated mutations inaccessible through rational design or directed evolution. Supporting this, Chen et al. applied ASR to resurrect four ancestral xylose isomerases (82–90% sequence identity to extant counterparts) and expressed them in recombinant *S. cerevisiae*. Remarkably, these strains achieved high intracellular xylose metabolic efficiency (Chen et al. 2023). Given ASR’s demonstrated capacity to enhance xylose-metabolizing enzymes, we hypothesize its application could identify novel xylose transporters with improved efficiency and glucose insensitivity.

In this study, we employed ASR to reconstruct ancestral xylose transporters (Xt). Xt3 and Xt7 were selected based on their phylogenetic positions, sequence divergence from modern proteins, and predicted structural competence, aligning with the goal of exploring ASR for discovering novel transporters. For comparative characterization, we used the engineered N326F^Xltr1p^ transporter as a positive control, as it represents a high-performance benchmark for xylose-specific transport (Jiang et al. 2020). Our results demonstrate that Xt3 exhibited superior xylose uptake activity under xylose-only conditions and mixed sugar compared to both N326F^XltR1p^ and Xt7. Crucially, during glucose-xylose co-fermentation, Xt3 significantly alleviated glucose-mediated inhibition of xylose uptake, thereby enhancing mixed-sugar utilization efficiency. Mechanistic studies through molecular docking and dynamics simulations further revealed that both ancestral transporters possess enhanced structural stability and higher substrate-binding affinity during xylose transport. This study represents the pioneering application of emerging biotechnological ASR in transporter engineering, showcasing its successful implementation.

## Results

### Ancestral sequence reconstruction and sequence analysis of ancestral xylose transporters

**Fig. 1:**
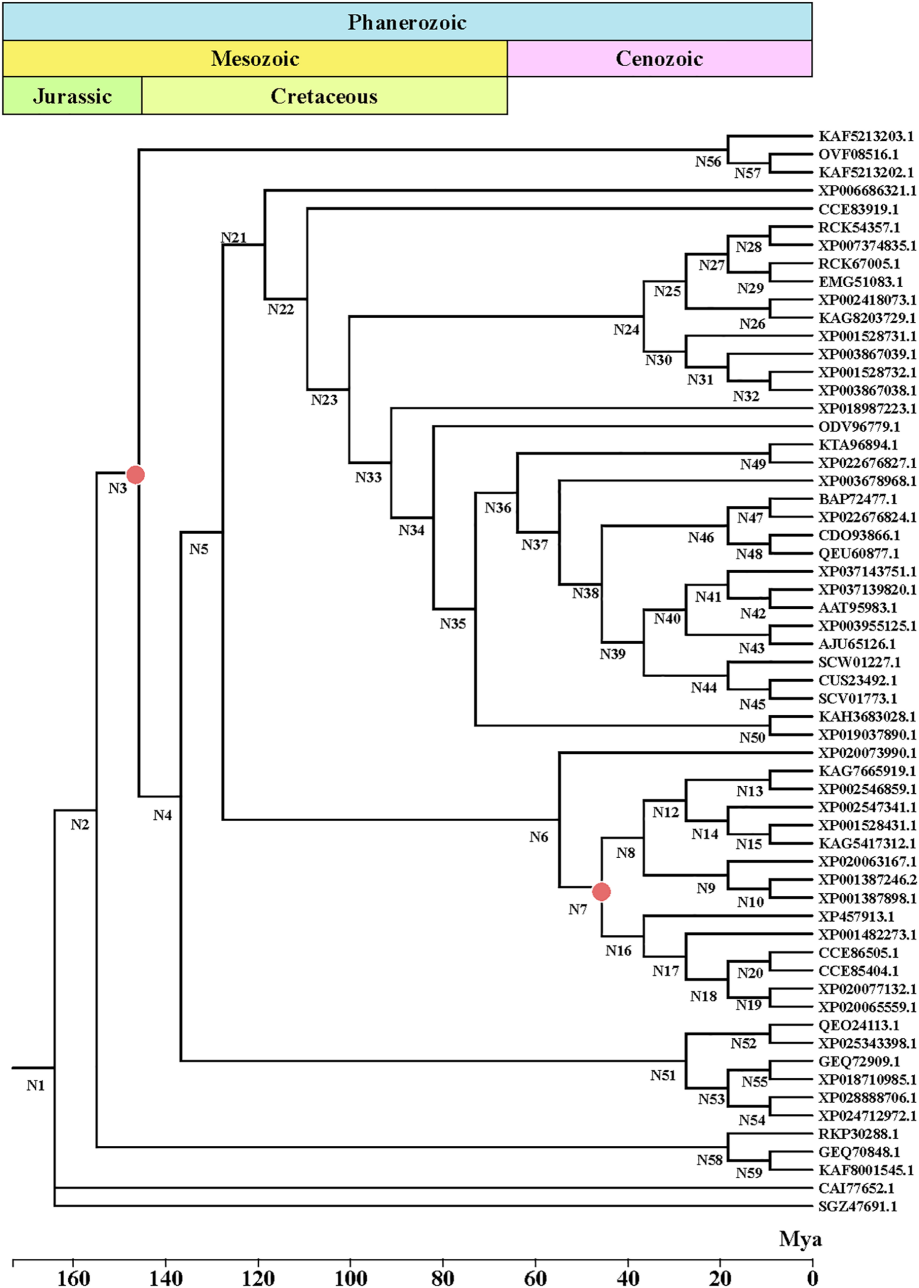
Uncorrelated relaxed clock chronogram for Gxf1 from *C. intermedia* and Sut1 from *S. stipitis*

Ancestral sequence reconstruction is an exceptionally potent methodology that can yield invaluable insights into the underlying determinants that govern protein structure, function, and evolution (Gumulya and Gillam 2017). To reconstruct ancestral xylose transporters with potentially enhanced function, we selected two benchmark heterologous transporters, Gxf1 from Candida intermedia and Sut1 from Scheffersomyces stipitis, as query sequences. These transporters were chosen based on their previously reported high efficiency in xylose uptake and expression in S. cerevisiae (Runquist et al. 2009; Kogje and Ghosalkar 2016), providing a strong functional foundation for ancestral reconstruction. Using the amino acid sequences of the xylose transporters proteins Gxf1 from *C. intermedia* and Sut1 from *S. stipitis* as query sequences, we retrieved homologous sequences via a BLASTP search against the NCBI protein database and obtained 500 homologous sequences. The 500 homologous sequences were processed using Cluster Database at High Identity with Tolerance (CD-HIT), a tool commonly used to remove duplicate and redundant sequences. This process resulted in the identification of 61 non-redundant homologous sequences, with pairwise identities ranging from 30 to 90%. The 61 non-redundant homologous sequences were aligned using MAFFT, and highly divergent regions in the alignment sequences were removed using MEGA (Supplementary Fig. 1). The optimal model of amino acid substitution in this alignment sequences was estimated to be LG + I + G by ProtTest. To construct a phylogenetic tree, we selected the maximum likelihood method with the chosen set of 61 non-redundant homologous sequences. Finally, by assigning the ancestral state with the highest-weighted posterior probability, we reconstructed the most likely ancestral sequences for each node on the tree. We date our phylogenetic tree using data from the Time Tree of Life (Fig. 1), select two ancestral nodes N3 and N7, designating them as ancestral xylose transporter 3 (Xt3) and ancestral xylose transporter 7 (Xt7) (Amino acid sequence, Supplementary Figs. 2 and 3). The Xt3 was estimated to be ~ 140 million years old and the Xt7 was estimated to be ~ 40 million years old. The sequence similarity between Xt3 and the existing sequences Gxf1 and Sut1 was 87.32% and 75.14%, respectively, while for Xt7 it was 81.89% and 78.36%. The mutations in the ancestral xylose transporters with respect to modern Gxf1 and Sut1 were distributed throughout the sequences (Supplementary Fig. 4). Subcellular localization and protein structure predictions indicated that both Xt3 and Xt7 were located in the cell membrane and were predicted to possess twelve transmembrane helices (Supplementary Fig. 5), a topology characteristic of sugar transporters in the Major Facilitator Superfamily (MFS). This structural feature supported their identity as potential membrane transporters, prompting further functional characterization.

### Subcellular localization of ancestral xylose transporters Xt3 and Xt7

**Fig. 2:**
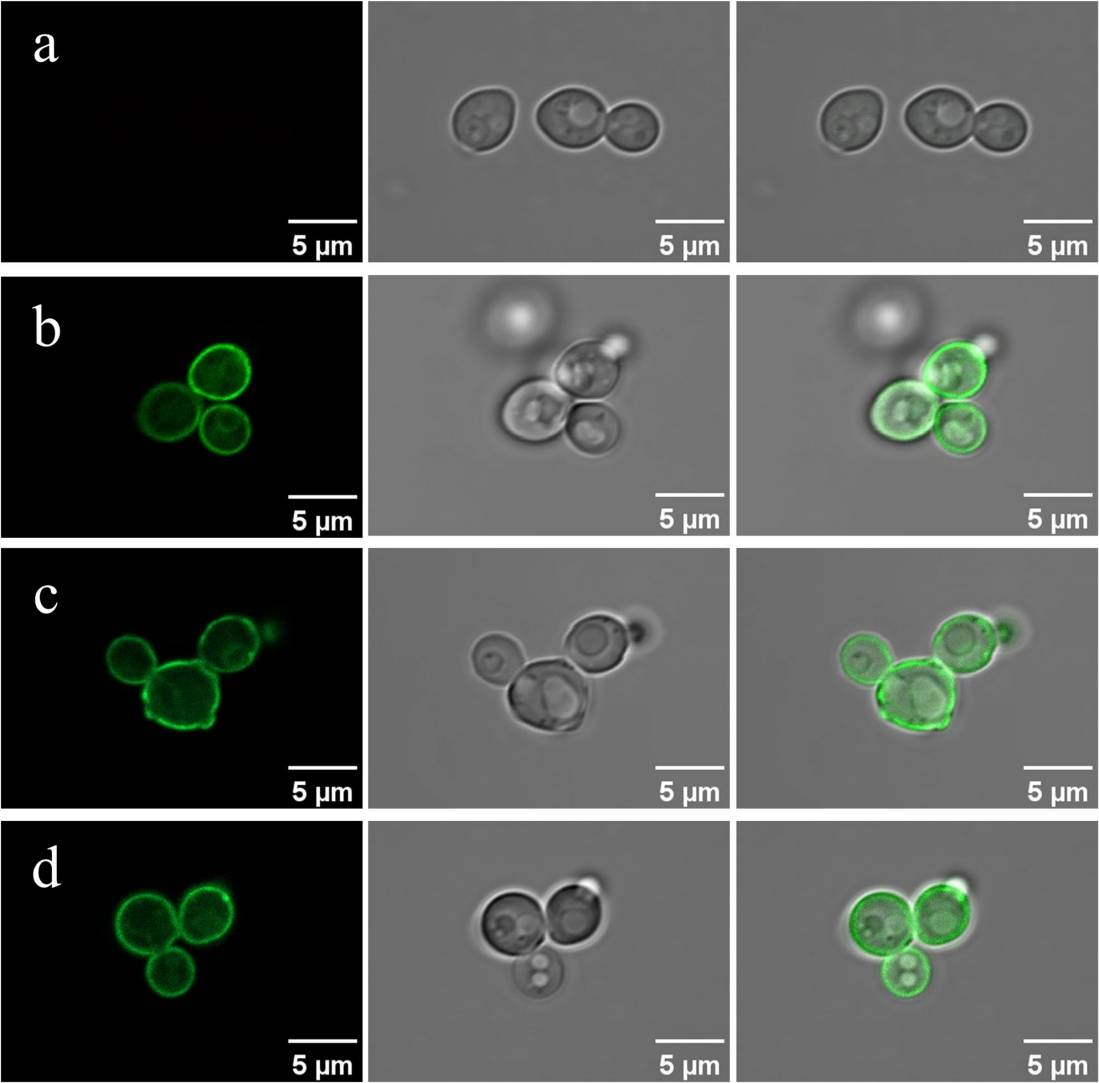
Subcellular localization based on the GFP fluorescence of xylose transporters. **a** Subcellular localization of the empty-vector control strain *S. cerevisiae* INVSc1-EV-pHM; **b** Subcellular localization of ancestral xylose transporter Xt3 in *S. cerevisiae*; **c** Subcellular localization of ancestral xylose transporter Xt7 in *S. cerevisiae*; **d** Subcellular localization of extant xylose transporter N326F^Xltr1p^ in *S. cerevisiae*, which was used as a positive control

The correct folding and localization of sugar transporters within the cell membrane are essential for their functionality as transmembrane proteins (Tjo and Conway 2024). In this study, we investigated the subcellular localization of ancestral xylose transporters Xt3 and Xt7 in *S. cerevisiae* by genetically fusing them with green fluorescent protein (GFP). Subsequently, confocal microscopy was used to capture fluorescence images of recombinant *S. cerevisiae* Xt3-GFP and recombinant *S. cerevisiae* Xt7-GFP (Fig. 2). As a positive control, recombinant *S. cerevisiae* N326F^Xltr1p^-GFP exhibited fluorescence on its cell membrane compared to wild-type *S. cerevisiae*. Notably, recombinant *S. cerevisiae* Xt3-GFP and recombinant *S. cerevisiae* Xt7-GFP displayed a distinct fluorescent halo at the periphery of the cell when compared with wild-type *S. cerevisiae*. These results demonstrated that ancestral sequences Xt3 and Xt7 were expressed and accurately localized in the cell membrane of *S. cerevisiae*.

### Functional characterization of ancestral xylose transporters Xt3 and Xt7

**Fig. 3:**
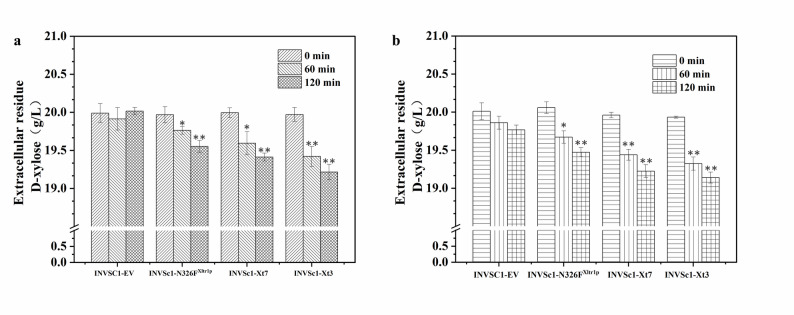
Characterization of xylose uptake ability of xylose transporters Xt3, Xt7, and N326F^Xltr1p^ in *S. cerevisiae*. **a** In a system with xylose as the sole carbon source, the extracellular xylose remnants of recombinant *S. cerevisiae* strains expressing Xt3, Xt7, or N326F^Xltr1p^. The *S. cerevisiae* INVSc1 with empty-vector, which lacks a functional xylose transporter, was included as a negative control. **b** In a mixed sugar system (xylose and glucose), the extracellular xylose remnants of the same set of recombinant strains. Wild-type INVSc1 was included as a negative control. Data are presented as mean values of triplicate experiments. The data was presented as the mean value ± SD of three biological replicates. Statistical comparisons between the values of samples and control were performed using a *t*-test. Significant differences were determined according to a threshold of *p < 0.05, **p < 0.01

The xylose uptake ability of recombinant *S. cerevisiae* overexpressing Xt3, Xt7, and N326F^Xltr1p^ in media with xylose as the sole carbon source was assessed by quantifying extracellular remnants of xylose using high-performance liquid chromatography (HPLC) (Jafari and Hematian Sourki 2024). Determining the extracellular remnants of xylose, the results showed that Xt3, Xt7 and N326F^Xltr1p^ exhibited high xylose uptake abilities. The xylose uptake ability of Xt3 was significantly higher than that of Xt7 and N326F^Xltr1p^ (Fig. 3a). Specifically, after 60 min of cultivation, the extracellular remnant of xylose in the recombinant *S. cerevisiae* INVSc1- N326F^Xltr1p^ was 1.46 times greater than that of *S. cerevisiae* INVSc1- Xt3. We further assessed the xylose uptake ability of recombinant *S. cerevisiae* overexpressing Xt3, Xt7, and N326F^Xltr1p^ in media with xylose and glucose by HPLC. The results showed that the ancestral xylose transporter Xt3 still exhibited stronger xylose uptake ability than N326F^Xltr1p^ in mixed sugars (Fig. 3b). Overall, these results demonstrate that the ancestral xylose transporters Xt3 and Xt7 mediate a faster and more efficient net uptake of xylose compared to the existing xylose transporter N326F^Xltr1p^.

### Comparison of xylose utilization

**Fig. 4:**
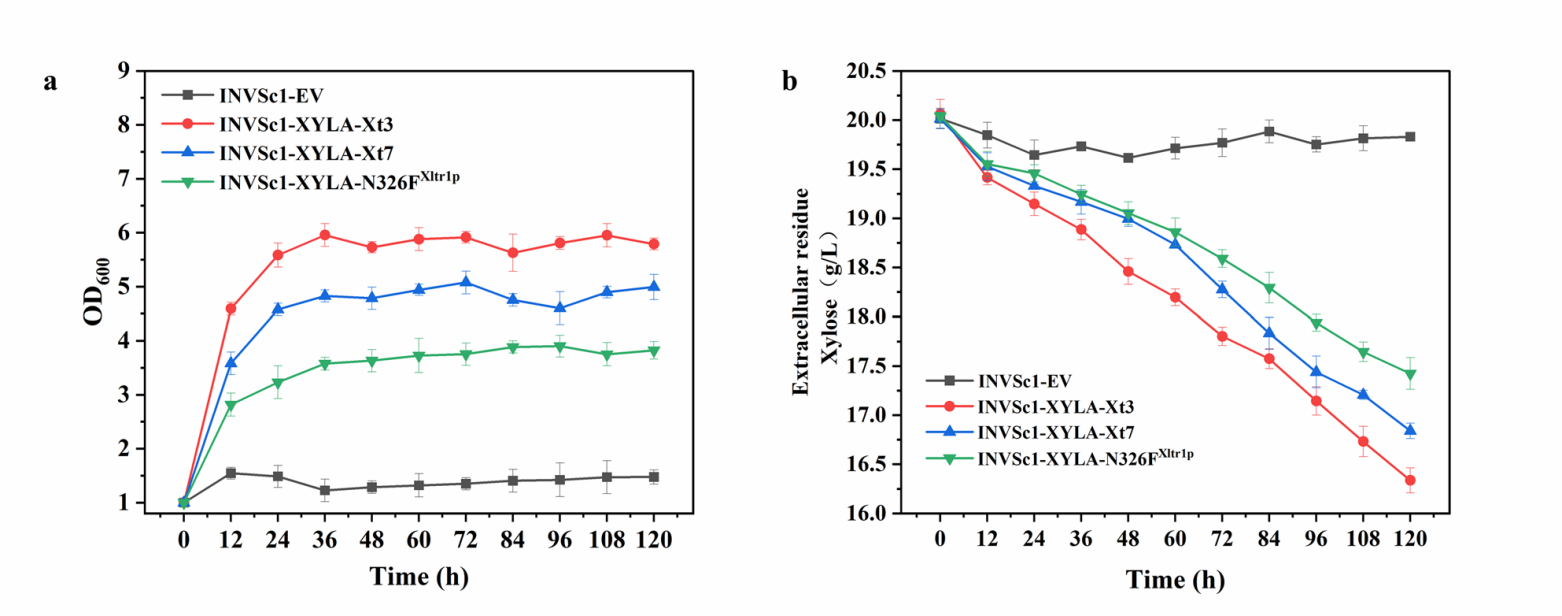
Growth curves (**a**) and xylose utilization capacity (**b**) of recombinant *S. cerevisiae* strains expressing both xylose isomerase and a xylose transporter (XYLA-Xt3, XYLA-Xt7, or XYLA-N326F^Xltr1p^) during fermentation with xylose as the sole carbon source. The *S. cerevisiae* INVSc1with empty-vector, which cannot metabolize xylose, was used as a negative control. The data was presented as the mean value ± SD of three biological replicates

To assess how the ancestral transporters influence overall xylose metabolism and cell growth, rather than just substrate uptake, we next constructed recombinant strains co-expressing a xylose transporter (Xt3, Xt7, or N326F^Xltr1p^) together with xylose isomerase (XYLA). This combination is necessary to enable the metabolic conversion of imported xylose into a utilizable carbon source, thereby allowing the measurement of xylose consumption and growth over an extended fermentation period. The efficiency of xylose utilization and the growth curve within 120 h in the medium with 20 g/L xylose were compared in the recombinant strains (Fig. 4). The results demonstrated that after 120 h, *S. cerevisiae* INVSc1 with empty-vector was practically unable to utilize xylose and showed no obvious growth, while the recombinant *S. cerevisiae* INVSc1-XYLA-Xt3 exhibited the highest xylose utilization and significant growth. Within 120 h of fermentation, *S. cerevisiae* INVSc1-XYLA-Xt3 consumed 3.66 g/L xylose, which was significantly higher than the consumption by *S. cerevisiae* INVSc1-XYLA-Xt7 (3.16 g/L, *p* < 0.05) and *S. cerevisiae* INVSc1-XYLA-N326F^Xltr1p^ (2.58 g/L, *p* < 0.01). These findings indicated that Xt3 and Xt7 possessed superior transport efficiencies over N326F^Xltr1p^, with Xt3 demonstrating enhanced xylose transport capabilities compared to Xt7. This implies that ancestral xylose transporters confer greater proficiency in xylose transportation, resulting in an accelerated rate of xylose utilization in the recombinant strains.

### Comparison of glucose/xylose co-utilization

**Fig. 5:**
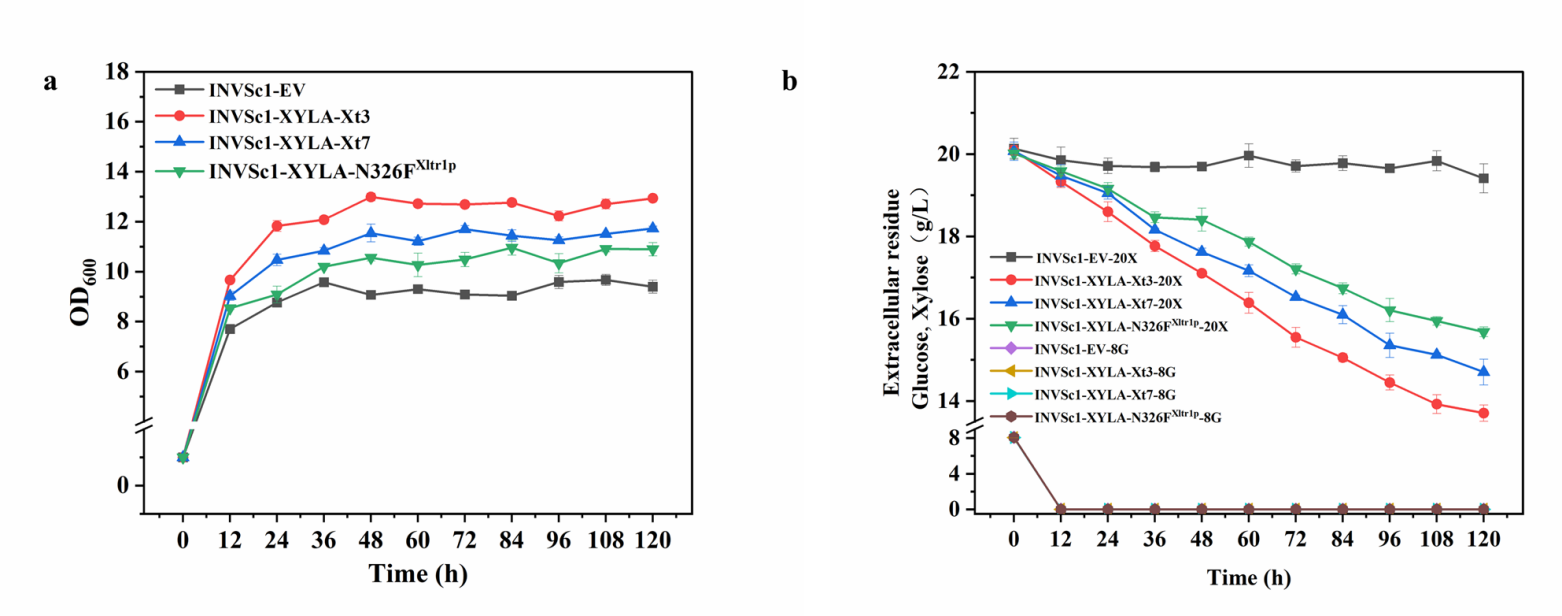
Growth curves (**a**) and sugar consumption (**b**) of recombinant *S. cerevisiae* strains expressing both xylose isomerase and a xylose transporter (XYLA-Xt3, XYLA-Xt7, or XYLA-N326F^Xltr1p^) during mixed sugar (20 g/L glucose and 8 g/L xylose) co-fermentation. The *S. cerevisiae* INVSc1 with empty-vector was used as a negative control for xylose consumption. The data was presented as the mean value ± SD of three biological replicates

The impact of ancestral xylose transporters on the co-consumption of xylose and glucose were further investigated in recombinant *S. cerevisiae* INVSc1-XYLA-Xt3, *S. cerevisiae* INVSc1-XYLA-Xt7, and *S. cerevisiae* INVSc1-XYLA-N326F^Xltr1p^. The kinetics of consumption of both sugars and the growth within 120 h during mixed sugar (20 g/L xylose and 8 g/L glucose) fermentation were studied (Fig. 5a, b). The growth profiles of the recombinant and wild-type strains during mixed-sugar co-fermentation were shown in Fig. 5a. The wild-type *S. cerevisiae* INVSc1 with empty-vector exhibited the slowest growth and the lowest final biomass density, consistent with its inability to utilize xylose after glucose depletion. All recombinant strains expressing a xylose transporter (Xt3, Xt7, or N326F^Xltr1p^) in conjunction with xylose isomerase (XYLA) demonstrated significantly improved growth kinetics and final optical density compared to the wild-type control. Among them, the strain expressing the ancestral transporter Xt3 (*S. cerevisiae* INVSc1-XYLA-Xt3) achieved the highest growth rate and maximal cell density, followed by the Xt7-expressing strain (*S. cerevisiae* INVSc1-XYLA-Xt7). The strain harboring the benchmark engineered transporter N326F^Xltr1p^ (*S. cerevisiae* INVSc1-XYLA-N326F^Xltr1p^) showed a clear growth advantage over the wild type but was outperformed by both ancestral transporter strains. These growth characteristics directly correlated with the respective xylose consumption profiles, indicating that the enhanced xylose transport capability conferred by the ancestral transporters, particularly Xt3, successfully translated into superior biomass generation during co-fermentation.

The sugar consumption profiles during co-fermentation were analyzed and were presented in Fig. 5b. As expected, glucose was rapidly and completely consumed by all *S. cerevisiae* strains within the first 12 h, significantly faster than xylose. The wild-type *S. cerevisiae* INVSc1 with empty-vector consumed glucose but was practically unable to utilize xylose, with minimal consumption observed throughout the fermentation. In contrast, all recombinant strains co-expressing XYLA and a xylose transporter exhibited sequential consumption of glucose and xylose. Quantitative analysis revealed that the recombinant *S. cerevisiae* INVSc1-XYLA-Xt3 strain achieved the highest total xylose consumption of 6.30 g/L, significantly surpassing the INVSc1-XYLA-Xt7 strain by 0.99 g/L (*p* < 0.05) and the INVSc1-XYLA-N326F^Xltr1p^ strain by 1.97 g/L (*p* < 0.01).

### Performance of ancestral xylose transporters under high-sugar condition

**Fig. 6:**
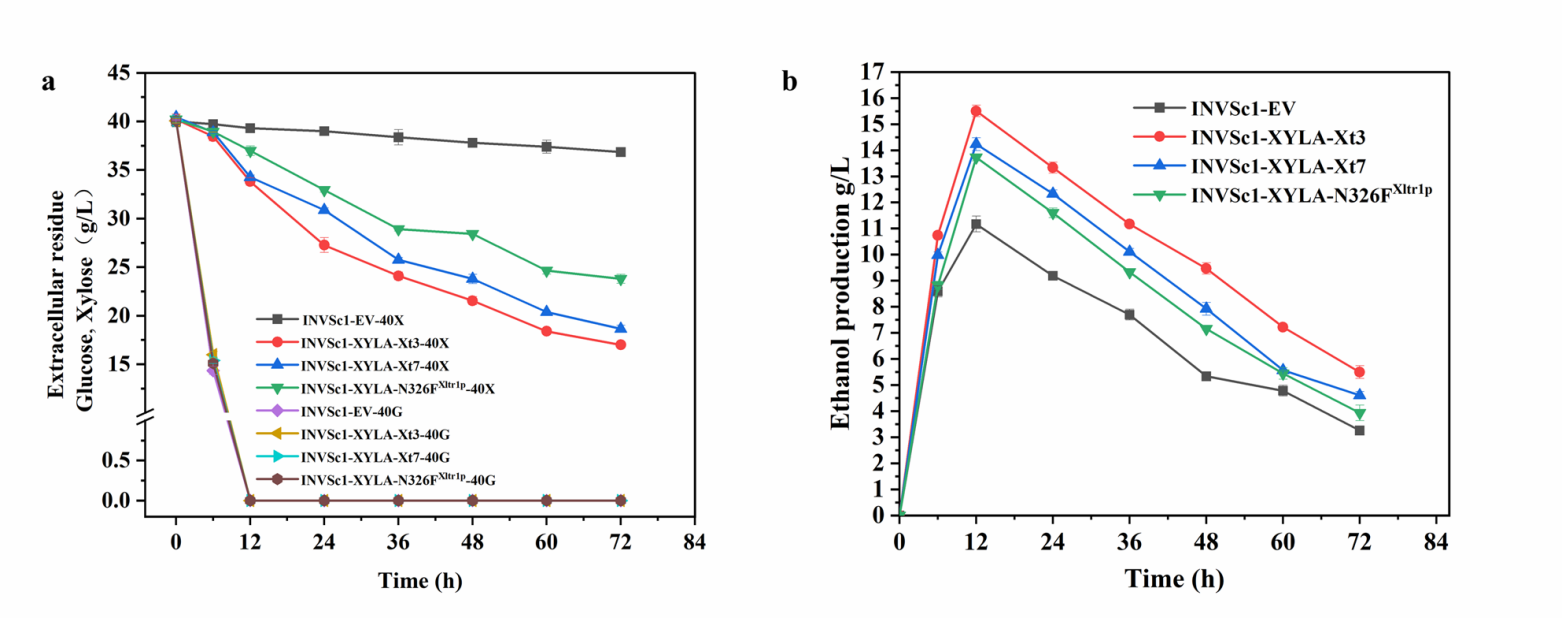
Sugar consumption (**a**) ethanol production (**b**) recombinant *S. cerevisiae* strains expressing both xylose isomerase and a xylose transporter (XYLA-Xt3, XYLA-Xt7, or XYLA-N326F^Xltr1p^) during mixed sugar (40 g/L glucose and 40 g/L xylose) co-fermentation. The *S. cerevisiae* INVSc1 with empty-vector was used as a negative control for xylose consumption. The data was presented as the mean value ± SD of three biological replicates

When glucose (40 g/L) and xylose (40 g/L) were supplied as cocarbon sources to mimic lignocellulosic hydrolysate, and the sugar consumption and ethanol production were studied (Fig. 6a, b). All the strains showed satisfactory performance with glucose consumed within 12 h. Quantitative analysis revealed that the recombinant *S. cerevisiae* INVSc1-XYLA-Xt3 strain achieved the highest total xylose consumption of 22.75 g/L, surpassing that of the INVSc1-XYLA-Xt7 (21.36 g/L) and INVSc1-XYLA-N326F^Xltr1p^ (16.22 g/L) strains. The ethanol production revealed that the recombinant *S. cerevisiae* INVSc1-XYLA-Xt3 strain achieved the highest total xylose consumption of 15.50 g/L, surpassing that of the INVSc1-XYLA-Xt7 (14.23 g/L) and INVSc1-XYLA-N326FXltr1p (13.72 g/L) strains. These results demonstrated that the ancestral xylose transporter Xt3 most effectively alleviated the glucose-mediated inhibition of xylose uptake, thereby enhancing the co-utilization efficiency of mixed sugars.

### Molecular docking and molecular dynamics simulation of ancestral xylose transporters

**Fig. 7:**
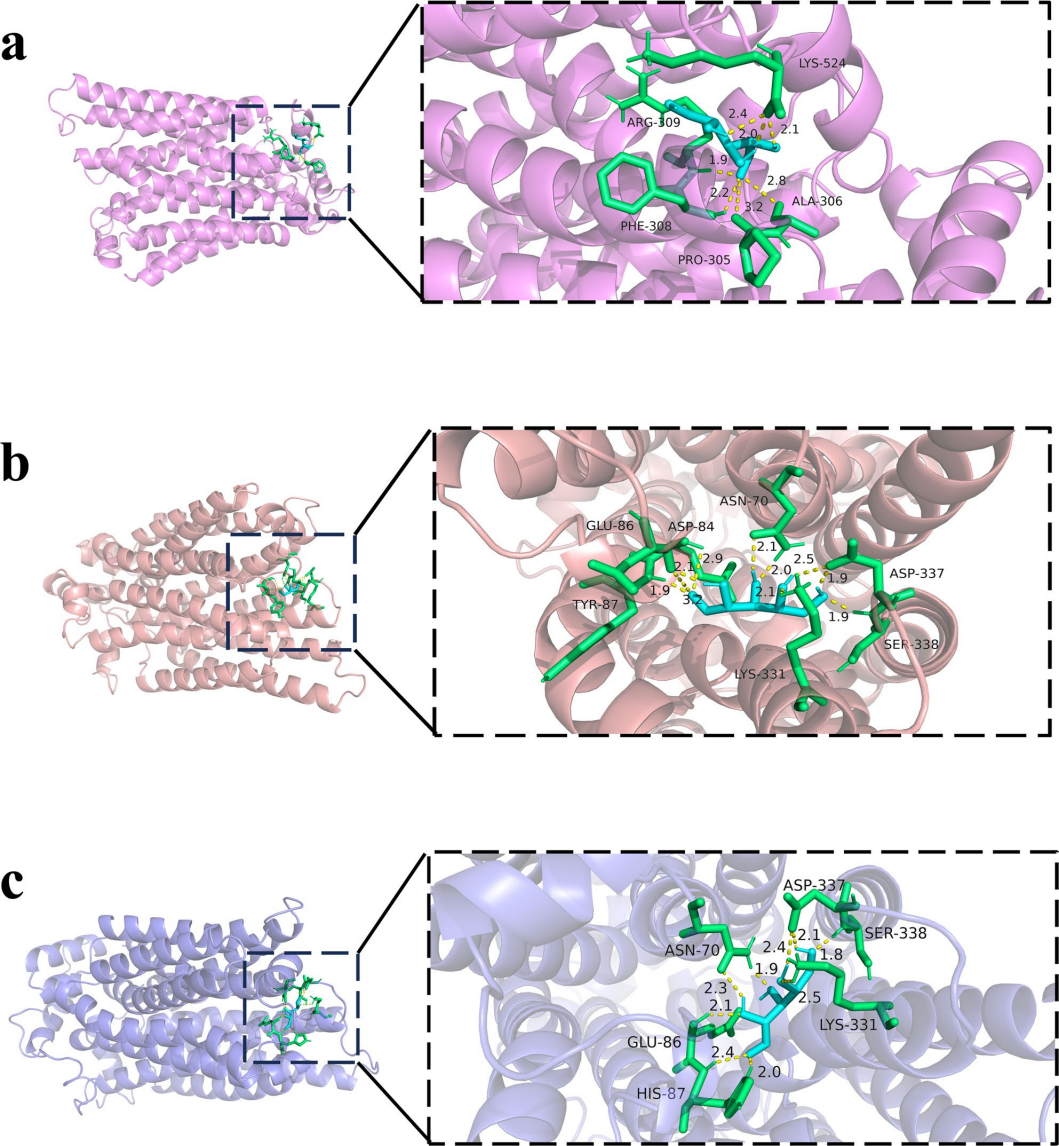
Molecular docking of ancestral xylose transporter Xt3, ancestral xylose transporter Xt7, and extant xylose transporter N326F^Xltr1p^

The experiments described above showed that the ancestral xylose transporter Xt3 exhibit higher uptake activity than the existing xylose transporter N326F^Xltr1p^. To elucidate the structural basis of this enhanced efficiency, we performed molecular docking of the xylose transporters with xylose (Luo et al. 2024). The binding affinities of the Xt3, Xt7, and N326F^Xltr1p^ with xylose were − 3.68 kcal/mol, − 3.32 kcal/mol, and − 3.15 kcal/mol, respectively (Fig. 7). While the observed difference in binding free energy between Xt3 and N326F^Xltr1p^ was numerically moderate (~ 0.53 kcal/mol), the consistently more favorable values for the ancestral transporters align with the enhanced functional performance observed in our uptake and fermentation assays. It should be noted that molecular docking provides a static and theoretical estimate; therefore, these results are interpreted as suggestive of a potentially optimized binding interaction rather than as conclusive evidence of dramatically altered affinity.

**Fig. 8:**
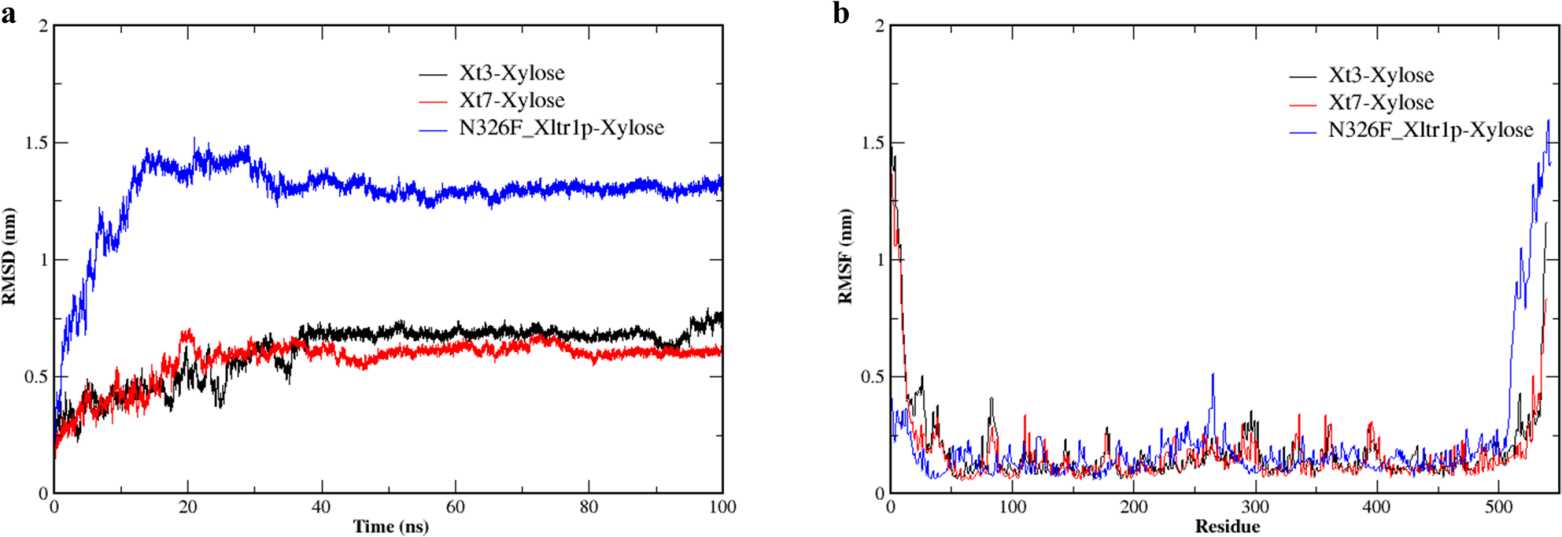
Molecular dynamics simulations of ancestral xylose transporter Xt3, ancestral xylose transporter Xt7, and extant xylose transporter N326F^Xltr1p^

Further, we performed a 100 ns molecular dynamics simulation (Ke et al. 2017) to investigate the tertiary structures of the Xt3, Xt7, and N326F^Xltr1p^, which elucidated alterations in their overall structural rigidity (Fig. 8). The root-mean-square deviation (RMSD), which measures the conformational change of a structure from its initial state, was analyzed to assess the structural rigidity of the transporters during simulation. A lower RMSD value typically indicates less structural fluctuation and greater conformational stability over time. The RMSD trajectories of Xt3, Xt7, and N326F^Xltr1p^ reached a stable plateau after 30 ns, 40 ns, and 40 ns, respectively. Analysis of the RMSD trajectories revealed critical dynamic differences: the Xt3-xylose and Xt7-xylose complexes not only reached a lower RMSD plateau (0.60 nm, 0.57 nm) but also demonstrated faster convergence and significantly greater trajectory stability throughout the simulation compared to the N326F^Xltr1p^ complex (Fig. 8a). The N326F^Xltr1p^ complex exhibited larger fluctuations and a higher, less stable plateau (1.27 nm), indicating greater structural flexibility. In contrast, the stable and convergent dynamic trajectory of Xt3 reflects its enhanced conformational stability during the simulation. This analysis was complemented by root-mean-square fluctuation (RMSF) measurements, which reflect residue-specific flexibility. The lower average RMSF values for Xt3 (0.18 nm) and Xt7 (0.17 nm) compared to N326^FXltr1p^ (0.22 nm) indicate reduced local flexibility and enhanced structural rigidity in the ancestral transporters (Fig. 8b). Together, the RMSD and RMSF results provide consistent evidence that the ancestral transporters, particularly Xt3, form more rigid and stable complexes with xylose, which may contribute to their improved transport efficiency observed in functional assays.

## Discussion

In recent years, numerous ancestral proteins and enzymes have been reconstructed with the primary aim of validating evolutionary hypotheses (Barkman 2024). However, the potential application of such reconstructions in biotechnology remains largely unexplored. Here, we employed ASR to reconstruct the ancestral xylose transporters relevant to biotechnology, and we focus on the xylose transport and the inhibitory effect of glucose on xylose transport.

The ancestral xylose transporter increased simultaneous utilization of xylose and glucose than existing xylose transporter N326F^Xltr1p^. Simultaneous utilization of xylose and glucose is limited due to the inhibition of xylose uptake by glucose (Saloheimo et al. 2007). To overcome this limitation, various putative genes, known heterologous xylose transporters, and engineered endogenous transporters have been expressed in *S. cerevisiae* over the past decade, while most of them showed preference for glucose over xylose (de Bueno et al. 2020; Reider Apel et al. 2016). In general, ancestral proteins are considered to be generalists having a broader range of applicability than existing proteins, which are considered specialist. In this study, the phenotype of efficient xylose uptake was captured by ASR and demonstrated by our Xt3. The ancestral proteins also exhibited other characteristics such as broad pH adaptability, higher expression yield, or non-specificity towards substrates (Perez-Jimenez et al. 2011). Thus, ASR emerge as a potential methodology for protein engineering with multiple applications in biotechnology, beyond its possible evolutionary implications (De Barruetabeña et al. 2019).

While this study provides comprehensive functional validation of the ancestral transporters, we acknowledge that it does not include the determination of classical kinetic parameters (Km and Vmax). Such measurements, typically requiring radiolabeled substrates and specialized instrumentation, were beyond the practical and budgetary scope of this project. However, the direct xylose uptake assays (Fig. 3a) and the performance in competitive co-fermentation (Figs. 5 and 6) serve as robust, physiologically relevant proxies for transport efficiency. These functional data unequivocally demonstrate a superior uptake rate for the ancestral transporters, particularly Xt3. Future work employing specialized host strains and techniques will aim to complement these findings with precise kinetic characterization.

In this study, the recombinant *S. cerevisiae* was constructed by expressing an ancestral xylose transporter to enhance xylose uptake across the cell membrane. As a result, the xylose consumption rate was improved compared to the wild-type strain. However, when compared with recombinant *S. cerevisiae* obtained through protein engineering of enzymes involved in xylose metabolism and evolution engineering, the xylose utilization rate of our recombinant *S. cerevisiae* was remained lower (Bae et al. 2021). This difference can be attributed to the fact that our approach focused solely on improving xylose transport without optimizing the intracellular metabolic flux for xylose assimilation. Therefore, subsequent improvement in intracellular xylose metabolism of recombinant *S. cerevisiae* can be achieved through protein engineering and evolution engineering. While this study focused on the critical upstream metric of sugar co-utilization efficiency to validate our transporter engineering strategy, the strong correlation between xylose consumption and ethanol production (Fig. 5 and Fig. 6) firmly establishes the functional success of the ancestral transporters. It is acknowledged that a comprehensive analysis of metabolic byproducts, such as glycerol and lactate, would provide deeper insights into the cellular metabolic state and potential redox imbalances. However, such an analysis falls within the realm of subsequent metabolic engineering of the host strain, which was beyond the transporter-centric scope of this work. Future studies will build upon these optimized transporters and employ detailed metabolomic profiling to further refine the entire metabolic network for maximizing yield and titer in industrial bioprocesses.

It is important to note that the functional characterization in this study was conducted in the laboratory strain *S. cerevisiae* INVSc1. This well-defined genetic background was chosen as an essential first step to provide a clear and unambiguous proof-of-concept, allowing for the precise attribution of the observed phenotypic improvements to the introduced ancestral transporters, free from the confounding genetic complexity of industrial strains. While we have demonstrated that the ancestral transporters possess intrinsic properties—such as enhanced uptake and reduced glucose inhibition—that are expected to be transferable, we acknowledge that validation in robust, industrially relevant yeast backgrounds (e.g., CEN.PK or Ethanol Red) is a critical next step. Such studies will be essential to fully assess the commercial potential and scalability of these engineered transporters in the context of actual bioprocessing conditions, and this constitutes a major focus of our ongoing and future work.

This study, in conjunction with recent advancements in protein engineering for the xylose pathway in *S. cerevisiae*, represents a progressive stride towards optimizing lignocellulosic biomass utilization. These findings underscore the pivotal role of molecular engineering of transport proteins in metabolic engineering (Sharma et al. 2018). Therefore, our research could serve as a valuable foundation for further enhancing xylose uptake through directed evolution, considering that existing proteins used as starting points often possess limited evolutionary capabilities due to their adaptation to specific functions via natural selection. Notably, successful laboratory evolution of pre-Cambrian enzymes has been reported recently, unveiling unexplored avenues for tackling more challenging targets (Gomez-Fernandez et al. 2018). Consequently, ancestral transporter reconstruction emerges as a versatile approach with diverse applications in protein engineering beyond its potential evolutionary significance within biotechnology.

## Conclusions

In this study, we demonstrated that the reconstructed ancient xylose transporters exhibit enhanced xylose uptake in recombinant *S. cerevisiae* and mitigate the inhibitory effect of glucose on xylose transport. Based on structure prediction and subcellular localization, it could be inferred that the ancestral xylose transporter maintains a similar fold to the existing xylose transporter and localized on the cell membrane. Molecular docking and molecular dynamics simulations provided insights into the structural rearrangement between the protein and substrate during reaction and integration stability. The results indicated that compared to the existing xylose transporter sequence, there was tighter binding between the ancestral xylose transporter Xt3 and xylose, resulting in a more stable overall conformation. Our findings redefine the paradigm of transporter engineering, emphasizing the untapped potential of ancestral protein resurrection for sustainable biomanufacturing.

## Materials and methods

### Strains, plasmids, medias, and chemicals

*Escherichia coli* DH5α was obtained from Vazyme Company and utilized as a host for DNA cloning and plasmid replication. *S. cerevisiae* INVSc1 was used as a host for testing the activities of Xt3, Xt7 and N326FXltr1p. The pMD18T-N326F^Xltr1p^ plasmid was donated by Professor Xu Fang of Shandong University and was used to amplify the mutant transporter gene (N326F^Xltr1p^, Genbank NO: ON260846.1) from *Trichoderma reesei* QM6a (ATCC 13631). The pMD18T-GFP plasmid was used to amplify the green fluorescent protein gene (GFP, Genbank NO: LN515608.1). The pUMRI-A plasmid was used for constructing recombinant plasmids carrying xylose transporter and xylose isomerase genes. The pHM368 expression vector was used for the expression of Xt3, Xt7 and N326F^Xltr1p^ in *S. cerevisiae*. All chemical reagents were purchased from Sigma-Aldrich (US) and Sinopharm Chemical Reagent Limited Company.

Yeast extract peptone dextrose (YPD) medium was used to culture *S. cerevisiae* INVSc1. Synthetic complete (SC) medium, which comprised 0.67% yeast-free amino nitrogen source, 2% glucose, 0.01% histidine (His), 0.01% leucine (Leu), and 0.01% tryptophan (Trp), was employed for screening recombinant *S. cerevisiae*. Additionally, Tris-X (containing 0.05 M Tris-HCl, 2% xylose at pH 5.0) and Tris-DX (composed of 0.05 M Tris-HCl, 2% xylose, and 0.8% glucose at pH 5.0) were used to characterize the uptake capacity of the xylose transporters. YPX (consisting of 1% yeast extract, 2% tryptone, and 2% xylose) and YP-DX (including 1% yeast extract, 2% tryptone, with both xylose and glucose at respective concentrations of 2% and 0.8%) were utilized to assess the utilization ability of recombinant *S. cerevisiae*. The maximum ethanol yield from 2% glucose and 0.8% g/L xylose reached 0.38 g/g.

### Alignment, phylogeny and ancestral sequence reconstruction

The general steps of ancestral sequence reconstruction (ASR) were performed as follows: the target protein was first identified based on prior studies (Runquist et al. 2009; Kogje and Ghosalkar 2016). Subsequently, homologous sequences of the target protein were retrieved using NCBI BLAST. A phylogenetic tree was constructed through multiple sequence alignment to infer the ancestral amino acid sequence at the designated ancestral node. Following the selection of this node, genetic engineering techniques were employed to reconstruct the ancestral amino acid sequence. The inferred sequence was reverse-translated into a nucleotide sequence via codon optimization, followed by artificial synthesis and cloning into an expression vector. Finally, gene expression in cell culture was carried out to revive ancient genes.

In this study, Gxf1 and Sut1 were selected as query proteins. Homologous amino acid sequences were identified in the National Center for Biotechnology Information (NCBI, https://www.ncbi.nlm.nih.gov/) database. Homologous sequences with more than 30% identity and over 70% coverage were downloaded to construct a dataset of homologous protein sequences. CD-HIT v4.8.1 was used to eliminate sequences that displayed significant discrepancies in amino acid length compared to the target proteins, as well as to remove highly similar sequences, resulting in a non-redundant sequence dataset. This non-redundant dataset was subjected to multiple sequence alignment (MSA) using MAFFT version 7 (Katoh and Standley 2013). The alignment results were visualized through MEGA for conservation analysis of homologous sequences (Tamura et al. 2007). The alignment was manually curated to remove sequences that compromised clarity, and non-conserved regions were corrected to improve gap reliability. The sequences were realigned using MAFFT, resulting in a refined multiple sequence alignment file. The final MSA file was used to determine the optimal amino acid replacement model through ProtTest v3.4.2 (Darriba et al. 2011, Le and Gascuel 2008). A systematic phylogenetic tree was constructed using the optimal amino acid substitution model implemented in IQ-TREE (von Nguyen et al. 2015), with robustness ensured through 1000 iterations of guided maximum likelihood (ML) estimation (Felsenstein 1981). The resulting phylogenetic tree was visualized using the Interactive Tree of Life online tool (https://itol.embl.de/) (Letunic and Bork 2021).

Ancestral sequences at each node were inferred using the FastML v3.11 program in the PAML software, based on the optimal amino acid substitution model and the final MSA file (Valle et al. 2014). The results were visualized using FigTree v1.4.3. Geological timelines for each ancestral sequence were predicted using TVBOT (https://www.chiplot.online/tvbot.html) (Kumar et al. 2017). Candidate representatives were identified by comparing ancestral sequences from the late Cretaceous period against the NCBI database, filtering those exhibiting over 90% similarity.

### Homology modeling, localization prediction, transmembrane structure prediction

SWISS-MODEL (https://swissmodel.expasy.org/) was used for homology modeling to predict the three-dimensional structures of ancestral xylose transporters Xt3 and Xt7 (de Waterhouse et al. 2018), and PyMOL was employed for visualizing the simulation results. WoLF PSORT (https://wolfpsort.hgc.jp/) was applied to determine the cellular localization of the predicted ancestral transporters Xt3 and Xt7 (Horton et al. 2007). Additionally, the DeepTMHMM online tool (https://dtu.biolib.com/DeepTMHMM) was used to predict the transmembrane domain architecture of ancestral transporters Xt3 and Xt7.

### Gene synthesis, plasmid construction, and Recombinant strain screening

The nucleotide sequences encoding inferred ancestral xylose transporters Xt3 and Xt7 were synthesized artificially (Generay Biotech Co., Ltd., Shanghai). The codon usage was automatically adapted to the codon bias of *S. cerevisiae* genes by GeneArt’s Web site service. The mutant of xylose transporter N326F^Xltr1p^ gene was amplified by PCR on pMD18T-N326F^Xltr1p^ plasmid using primers (N326F^Xltr1p^-F: 5-CTCAGCACAAGTGGTACCAATACTTCA-3 and N326F^Xltr1p^-R: 5-CCGAGCGTGAAAGGATTTGCC-3). The GFP gene was amplified by PCR on pMD18T-GFP plasmid using primers (GFP-F: 5-ATGCGTTTCTCCGAGAAGCTCG.

−3 and GFP-R: 5-CCGAGCGTGAAAGGATTTGCC-3). The fusion of N326F^Xltr1^, Xt3, and Xt7 with GFP were constructed by overlap extension PCR to obtain N326F^Xltr1p^-GFP, Xt3-GFP, and Xt7-GFP. Finally, the N326F^Xltr1p^, Xt3, Xt7, N326F^Xltr1p^-GFP, Xt3-GFP, and Xt7-GFP were constructed on the pHM368-pgk vector using ClonExpress II One Step Cloning Kit to obtain the recombinant plasmids pHM368-pgk-N326F^Xltr1p^, pHM368-pgk-Xt3, pHM368-pgk-Xt7, pHM368-pgk-N326F^Xltr1p^-GFP, pHM368-pgk-Xt3-GFP, and pHM368-pgk-Xt7-GFP. The N326F^Xltr1p^, Xt3, and Xt7 were constructed on the pUMR-XYLA vector using ClonExpress II One Step Cloning Kit to obtain the recombinant plasmids pUMR-XYLA-N326F^Xltr1p^, pUMR-XYLA-Xt3, and pUMR-XYLA-Xt7.

Recombinant plasmid pHM368-pgk-N326F^Xltr1p^, pHM368-pgk-Xt3, pHM368-pgk-Xt7, pHM368-pgk-N326F^Xltr1p^-GFP, pHM368-pgk-Xt3-GFP, and pHM368-pgk-Xt7-GFP and were linearized by *Hpa* I and transformed into *S. cerevisiae* INVSc1 by electroporation. Recombinant plasmid pUMR-XYLA-N326F^Xltr1p^, pUMR-XYLA-Xt3, and pUMR-XYLA-Xt7 were linearized by *Sfi* I and transformed into *S. cerevisiae* INVSc1 by electroporation. The electroporation was performed as our previously research described (Huang et al. 2024). Recombinant *S. cerevisiae* was screened by SC-Ura, and with polymerase chain reaction (PCR) verification for target genes of N326F^Xltr1p^, Xt3, and Xt7, obtained recombinant *S. cerevisiae* INVSc1-Xt3, *S. cerevisiae* INVSc1-Xt7, *S. cerevisiae* INVSc1-N326F^Xltr1p^, *S. cerevisiae* INVSc1-Xt3-GFP, *S. cerevisiae* INVSc1-Xt7-GFP, *S. cerevisiae* INVSc1-N326F^Xltr1p^-GFP, *S. cerevisiae* INVSc1 XYLA-N326F^Xltr1p^, *S. cerevisiae* INVSc1 XYLA-Xt3, and *S. cerevisiae* INVSc1 XYLA-Xt7. To construct the empty-vector control strains, the pHM368-pgk and pUMR vectors were linearized using the restriction enzymes *Hpa* I and *Sfi* I, respectively. The linearized vectors were then independently transformed into *S. cerevisiae* INVSc1 by electroporation, yielding the control strains *S. cerevisiae* INVSc1-EV-pHM and *S. cerevisiae* INVSc1-EV-pUMR.

### Subcellular localization of xylose transporters

Transporters fused to GFP were expressed in *S. cerevisiae* to determine their subcellular localization. Recombinant *S. cerevisiae* INVSc1-Xt3-GFP, *S. cerevisiae* INVSc1-Xt7-GFP and *S. cerevisiae* INVSc1-N326F^Xltr1p^-GEP were cultured in YP-DX medium for 12 h and harvested and then resuspended in 50 mmol/L phosphate buffered saline (PBS, pH 6.5). Cells were spotted onto microscope slides, and images were obtained using Laser Scanning Confocal Microscopy (Zeiss, LSM 710).

### Determination of the xylose uptake capacity of xylose transporters

*S. cerevisiae* INVSc1 and recombinant strains *S. cerevisiae* strains INVSc1-Xt3, *S. cerevisiae* INVSc1-Xt7, and *S. cerevisiae* INVSc1-N326F^Xltr1p^ were activated on YPD agar plates. Individual colonies were transferred into 10 mL of YPD medium and incubated at 28 °C with shaking at 200 rpm for 24 h. Subsequently, 2 mL of the seed culture was inoculated into 200 mL of fresh YPD medium and incubated under the same conditions for an additional 12 h. The cultures were centrifuged at 1000 g for 2 min, and the pellet was washed three times with sterile water. The pellet was resuspended in 1 mL sterile water and incubated for another 24 h to deplete intracellular glucose reserves. The cells were then inoculated into SC-X media, and samples were collected at 0, 30, 60, 90, 120, 150, and 180 min. The supernatant obtained after centrifugation was filtered through a 0.22 μm filter membrane, and the extracellular xylose content was determined by HPLC. The pellet was resuspended in 1 mL sterile water and incubated at 37 °C for 12 h to facilitate the release of intracellular xylose, which was subsequently analyzed using HPLC.

### Evaluation of xylose utilization capacity of Recombinant *S. cerevisiae* strains

*S. cerevisiae* INVSc1 and recombinant strains INVSc1 XYLA-Xt3, INVSc1 XYLA-Xt7, and INVSc1 XYLA-N326F^Xltr1p^ were activated on YPD agar plates. Individual colonies were transferred into 10 mL of YPD medium and incubated at 28 °C with shaking at 200 rpm for 24 h. Then, 1 mL of the seed culture was inoculated into 100 mL of fresh YPD medium and incubated under the same conditions for an additional 12 h. The cultures were centrifuged at 1000 g for 2 min, and the pellet was washed three times with sterile water. The recombinant strains were resuspended in 5 mL sterile water and transferred to 50 mL of YPX or YPDX medium. Samples were collected every 12 h, and the absorbance at OD_600_ was measured using a UV–Vis spectrophotometer. The intracellular and extracellular xylose levels of the recombinant *S. cerevisiae* strains were determined by HPLC.

### HPLC

Glucose, xylose and ethanol were analyzed by a high-performance liquid chromatography (HPLC) system. All samples were centrifuged at 16,000 g and room temperature for 3 min and then filtered through a 0.22 μm acetic acid fiber filter prior to injection. The high-performance liquid chromatography system (HPLC, Agilent 1260 Infinity II, Germany) was equipped with a refractive index detector (RID, Agilent 1260 G7162A, Germany) and an Aminex HPX-87 H column (300 mm × 7.8 mm, Bio-Rad, Hercules, CA). The column temperature was set at 55 °C, and the RID detector was operated at 30 °C. The mobile phase consisted of 5 mM H₂SO₄ with a flow rate of 0.5 mL min⁻¹ and an injection volume of 10 µL.

### Molecular docking and molecular dynamics simulation

AlphaFold3 was used to predict the structures of Xt3, Xt7, and N326FXltr1p. ChemDraw software was employed to illustrate the structure of the xylose substrate. Molecular docking with xylose was performed using AutoDock Tools (http://autodock.scripps.edu/resources/adt), and the results were visualized using PyMOL (https://www.pymol.org) to analyze protein-small molecule interactions. Molecular dynamics simulations (MD) were conducted using GROMACS 5.0.2 software with the AMBE99SB force field. The procedure involved isolating xylose and its docking complexes with Xt3, Xt7, and Xltr1p. Protein topology and hydration structures were generated using the TIP3P water model from the AMBE99SB force field. Hydrogen atoms were added to xylose, and a compatible hydration structure was generated using acpype (https://www.bio2byte.be/acpype/submit/). The protein and small molecule hydration structures were merged, ensuring a distance of 10 Å between solute molecules and the surrounding box. An equivalent number of water molecules were replaced with negative and positive ions to achieve charge neutrality within the system. Residue protonation states were established based on pH 7.0. Energy minimization was performed using the steepest descent algorithm for 10,000 steps, followed by a 100 ns molecular dynamics simulation at 301.15 K and atmospheric pressure of 1.01 bar. Binding free energy was calculated from MD simulation trajectories using MM/PBSA analysis in GROMACS, along with energy decomposition assessments.

### Statistical analysis

Superior Performance Software Systems (SPSS) was used for statistical analyses. All experiments about xylose and glucose fermentation analysis were carried out in triplicate. From the initial three duplicate measurements, the mean values were estimated. Statistical comparisons in other experiment between two values were performed with a *t*-test, and significant differences were determined according to a threshold of *p < 0.05; **p < 0.01.

## Supplementary Information

Supplementary Material 1

## Data Availability

The datasets used and/or analysed during the current study are available from the corresponding author on reasonable request.
