# Supplementary material for: Resurrection and characterization of ancestral xylose transporters enhance the capability of xylose uptake in the mixed sugar co-fermentation of Recombinant Saccharomyces cerevisiae
Source: Bioresour Bioprocess. 2026 Jan 5;13(1):1. doi: 10.1186/s40643-025-00995-1 (PMC12770209; doi:10.1186/s40643-025-00995-1)
Supplement: Supplementary file 1 — Supplementary Material 1 [file 40643_2025_995_MOESM1_ESM.docx]

**Supplementary materials**

**Title: Resurrection and characterization of ancestral xylose transporters enhance the capability of xylose uptake in the mixed sugar co-fermentation of recombinant *Saccharomyces cerevisiae***

**Authors:** Peining Zhang^1^, Zhaoqing He^1^, Zhengbing Jiang^1*^, Huanan Li^1*^

^1^State Key Laboratory of Biocatalysis and Enzyme Engineering, School of Life Sciences, Hubei University, Wuhan 430062, PR China

* Corresponding author: Zhengbing Jiang

School of Life Science, Hubei University

Email: [zhbjiang@hubu.edu.cn](mailto:zhbjiang@hubu.edu.cn)

Tel: +86 (27) 88663882-3026;

* Corresponding author: Huanan Li

School of Life Science, Hubei University

Email: [huananli@hubu.edu.cn](mailto:huananli@hubu.edu.cn)

Tel: +86 (27) 88663882-3026;


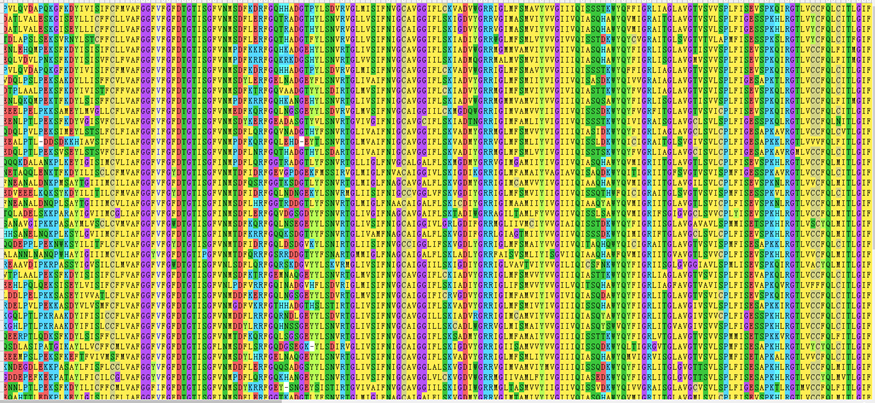
 Fig. S1 Multiple sequence alignment of 61 homologous sequences


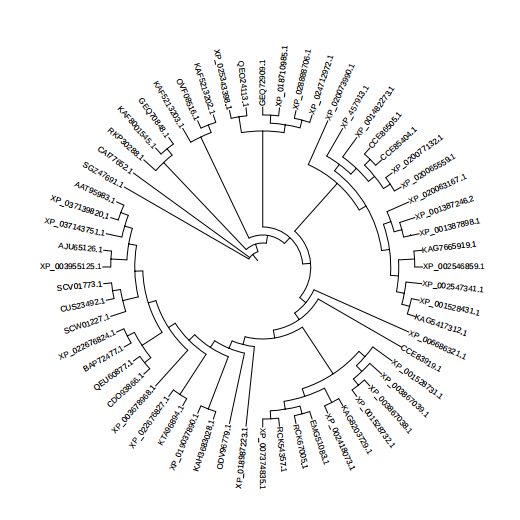


Fig. S2 系统发育树

MSQENTSSGATTPVNGSILEKEKEDEENLLPQLPEKSFKDYIVISIFCFLVAFGGFVFGFDTGTISGFVNMSDFKRRFGQHNADGEHYLSNVRTGLIVSIFNVGCAVGGIFLSKIADVWGRRIGLMFSMVIYVVGIIIQIASQHKWYQYFIGRLISGLAVGTVSVVSPLFISEVSPKQIRGTLVCCFQLCITLGIFLGYCTTYGTKTYTDSRQWRIPLGLCFAWAIFLVVGMLSMPESPRYLVEKNRIDEAKKSIARSNKVSEEDPAVYTEVQLIQAGIDREALAGKASWKELITGKPKIFRRVIMGIMLQSLQQLTGDNYFFYYGTTIFKAVGLKDSFQTSIILGIVNFASTFVGIYAIERMGRRMCLLTGSACMFVCFIIYSLLGSQHLYKHGYSSNTRKPTGNAMIFITCLYIFFFASTWAGGVYCIVSESYPLRIRSKAMSVATAANWMWGFLISFFTPFITSAIHFYYGFVFTGCLLFSFFYVYFFVYETKGLSLEEVDELYASGTLPWKSAGWVPPSVEHMAHSTGYADKPTD

ATGTCCCAGGAGAACACAAGCAGCGGTGCCACCACACCCGTTAACGGCAGCATTTTGGAGAAGGAAAAGGAGGACGAGGAGAACTTGCTGCCACAATTGCCAGAAAAGTCCTTCAAGGACTACATCGTCATCTCCATCTTCTGCTTCCTGGTCGCTTTCGGTGGTTTTGTTTTCGGTTTCGACACCGGTACCATCAGTGGTTTTGTCAACATGTCCGACTTCAAGAGGAGGTTCGGTCAACACAACGCCGATGGTGAACATTACCTGTCTAACGTCAGGACGGGTTTGATCGTATCCATCTTCAACGTCGGCTGCGCTGTTGGTGGAATTTTCTTGTCTAAGATCGCTGACGTCTGGGGTAGGAGAATTGGTTTGATGTTCTCCATGGTCATCTACGTCGTCGGTATCATCATCCAGATCGCTTCTCAGCACAAGTGGTACCAATACTTCATCGGCAGGCTGATCTCCGGTTTGGCTGTTGGTACTGTTTCCGTTGTCTCCCCATTGTTCATCTCCGAAGTCTCTCCAAAGCAGATCAGGGGTACCTTGGTCTGTTGTTTCCAGTTGTGCATCACCCTGGGCATTTTCTTGGGTTACTGCACTACCTACGGTACCAAGACTTACACCGACTCTAGGCAATGGAGGATCCCATTGGGTTTGTGTTTCGCTTGGGCTATCTTCCTGGTCGTTGGAATGTTGTCCATGCCTGAGTCTCCTAGATACCTGGTTGAGAAGAACAGGATCGACGAGGCTAAGAAGAGCATCGCTAGGTCTAACAAGGTCTCCGAAGAGGACCCTGCTGTTTATACGGAAGTGCAACTGATCCAGGCCGGTATAGATAGAGAGGCACTAGCAGGTAAGGCCAGTTGGAAAGAACTGATCACGGGTAAGCCCAAGATCTTCAGAAGGGTTATCATGGGCATCATGCTGCAGTCACTGCAACAACTAACCGGAGATAACTACTTCTTCTACTACGGTACCACCATCTTCAAGGCCGTCGGTTTGAAAGACAGCTTCCAAACATCCATCATCCTGGGTATCGTCAACTTCGCTTCCACTTTCGTCGGAATCTACGCTATCGAGAGGATGGGCAGGAGAATGTGCTTGTTGACTGGTTCTGCCTGCATGTTCGTCTGTTTCATCATCTACTCCCTGCTGGGTTCCCAGCATTTGTACAAACACGGTTACTCCTCCAACACCAGGAAGCCAACTGGTAATGCTATGATCTTCATCACCTGCCTGTACATCTTCTTCTTCGCCTCTACCTGGGCTGGTGGTGTTTATTGTATCGTCTCCGAGTCGTACCCCCTTAGAATAAGGTCAAAGGCCATGTCCGTCGCTACCGCTGCCAATTGGATGTGGGGTTTTTTGATCTCCTTCTTCACCCCCTTCATCACCAGCGCTATTCATTTCTACTACGGATTCGTCTTCACCGGCTGTCTACTATTCTCCTTCTTCTACGTCTACTTCTTCGTCTACGAGACCAAGGGCTTGTCTCTTGAGGAAGTTGACGAGTTGTACGCCTCTGGTACTTTGCCATGGAAGTCTGCCGGTTGGGTTCCTCCTTCTGTTGAGCATATGGCTCACTCTACCGGCTATGCTGATAAGCCTACTGACTAG

Fig. S2 Amino acid sequence and nucleotide sequence of the ancestral xylose transporter Xt3

MSQQELSSGVQTPVNSSILEKDEEQIQDQLPTLPEKSFKDYILISFFCLLVAFGGFVFGFDTGTISGFVNMSDFKQRFGQLNADGEYYLSNVRTGLIVSIFNVGCAVGGIFLSKVADVYGRRIGLMFSMVIYVVGIIVQISSQDKWYQIFIGRAITGLAVGTVSVLSPLFISESSPKQLRGTLVCCFQLCITLGIFLGYCTTYGTKTYSDSRQWRIPLGLCFAWAIMLVVGMVCMPESPRYLVEKNRIEDAKKSIARSNKVSPEDPAVYTEVQLIQAGIDREKLAGSASWTELITGKPKIFRRVIMGIMLQSLQQLTGDNYFFYYGTTIFKAVGLKDSFQTSIILGVVNFASTFVGIYAIERFGRRLCLLTGSACMFVCFIIYSVLGSVHLYKDGYDGPTYKPTGNAMIFITCLYIFFFASTWAGGVYCIVSETYPLRIRSKAMAVATAANWMWGFLISFFTPFITSAIHFYYGFVFTGCLLFSFFYVYFFVYETKGLSLEEVDELYAQGVLPWKSSSWVPPSKEQMAHSTGYAAKPED

ATGTCCCAACAAGAATTGTCCTCCGGTGTTCAAACCCCAGTTAACTCCTCCATTTTGGAAAAGGATGAAGAACAAATTCAAGATCAATTGCCAACCTTGCCAGAAAAGTCCTTCAAGGATTACATTTTGATTTCCTTCTTCTGTTTGTTGGTTGCTTTCGGTGGTTTCGTTTTCGGTTTCGATACCGGTACCATTTCCGGTTTCGTTAACATGTCCGATTTCAAGCAAAGATTCGGTCAATTGAACGCTGATGGTGAATACTACTTGTCCAACGTTAGAACCGGTTTGATTGTTTCCATTTTCAACGTTGGTTGTGCTGTTGGTGGTATTTTCTTGTCCAAGGTTGCTGATGTTTACGGTAGAAGAATTGGTTTGATGTTCTCCATGGTTATTTACGTTGTTGGTATTATTGTTCAAATTTCCTCCCAAGATAAGTGGTACCAAATTTTCATTGGTAGAGCTATTACCGGTTTGGCTGTTGGTACCGTTTCCGTTTTGTCCCCATTGTTCATTTCCGAATCCTCCCCAAAGCAATTGAGAGGTACCTTGGTTTGTTGTTTCCAATTGTGTATTACCTTGGGTATTTTCTTAGGTTACTGTACCACCTACGGTACCAAGACCTACTCCGATTCCAGACAATGGAGAATTCCATTGGGTTTGTGTTTCGCTTGGGCTATTATGTTGGTTGTTGGTATGGTTTGTATGCCAGAATCCCCAAGATACTTGGTTGAAAAGAACAGAATTGAAGATGCTAAGAAGTCCATTGCTAGATCCAACAAGGTTTCCCCAGAAGATCCAGCTGTTTACACCGAAGTTCAATTGATTCAAGCTGGTATTGATAGAGAAAAGTTGGCTGGTTCCGCTTCCTGGACCGAATTGATTACCGGTAAGCCAAAGATTTTCAGAAGAGTTATTATGGGTATTATGTTGCAATCCTTGCAACAATTGACCGGTGATAACTACTTCTTCTACTACGGTACCACCATTTTCAAGGCTGTTGGTTTGAAGGATTCCTTCCAAACCTCCATTATTTTGGGTGTTGTTAACTTCGCTTCCACCTTCGTTGGTATTTACGCTATTGAAAGATTCGGTAGAAGATTGTGTTTGTTGACCGGTTCCGCTTGTATGTTCGTTTGTTTCATTATTTACTCCGTTTTGGGTTCCGTTCATTTGTACAAGGATGGTTACGATGGTCCAACCTACAAGCCAACCGGTAACGCTATGATTTTCATTACCTGTTTGTACATTTTCTTCTTCGCTTCCACTTGGGCTGGTGGTGTTTACTGTATTGTTTCCGAAACCTACCCATTGAGAATTAGATCCAAGGCTATGGCTGTTGCTACCGCTGCTAACTGGATGTGGGGTTTCTTGATTTCCTTTTTCACCCCATTCATTACCTCCGCTATTCATTTCTACTACGGTTTCGTTTTTACCGGTTGTTTGTTGTTCTCCTTCTTCTACGTTTACTTCTTCGTTTACGAAACCAAGGGTTTGTCCTTGGAAGAAGTTGATGAATTGTACGCTCAAGGTGTTTTGCCATGGAAGTCCTCCTCCTGGGTTCCTCCTTCTAAGGAACAAATGGCTCATTCCACCGGTTACGCTGCTAAGCCAGAAGATTAG

Fig. S3 Amino acid sequence and nucleotide sequence of the ancestral xylose transporter Xt7

Fig.
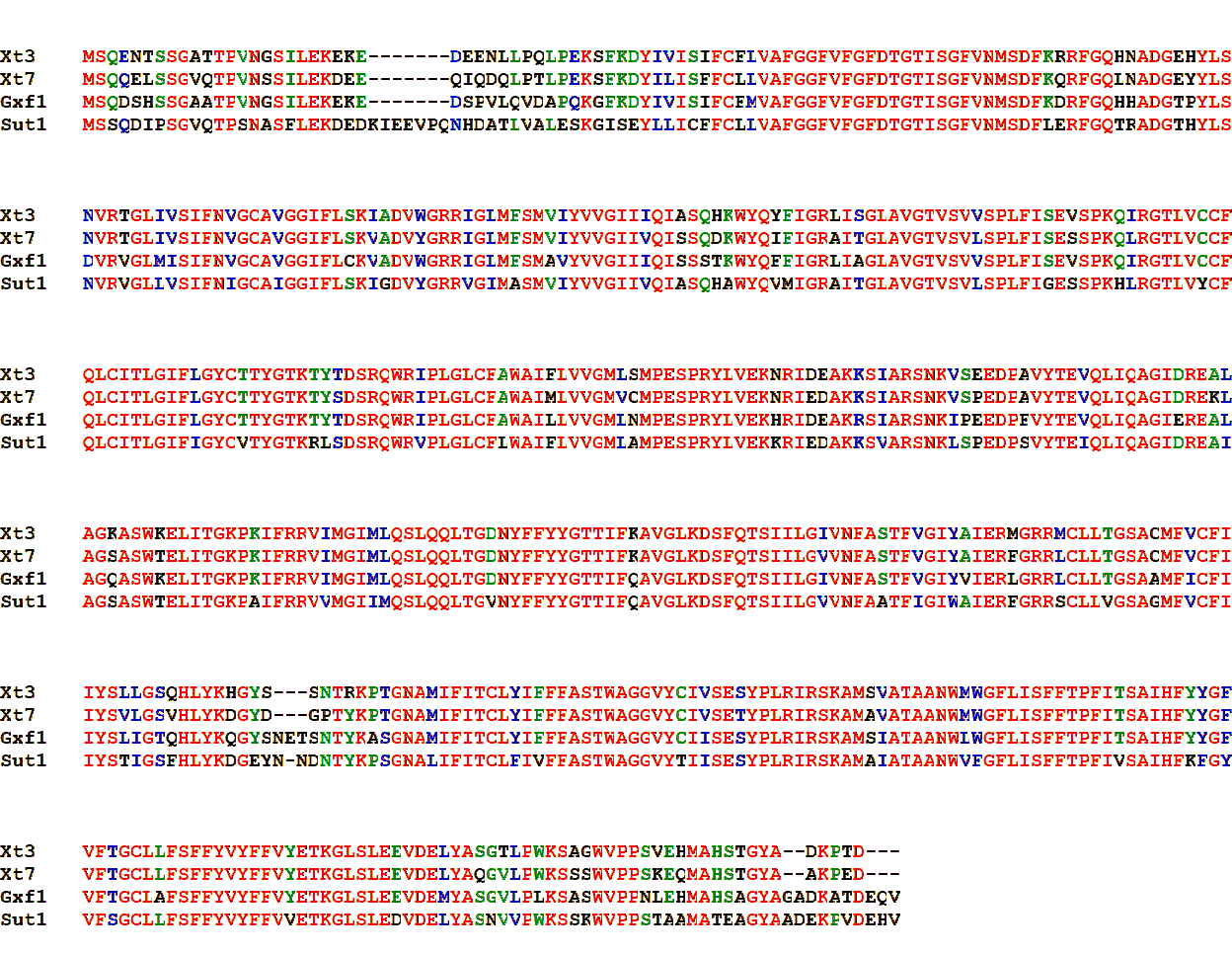
S4 Sequence comparison of Xt3, Xt7, Gxf1 and Sut1


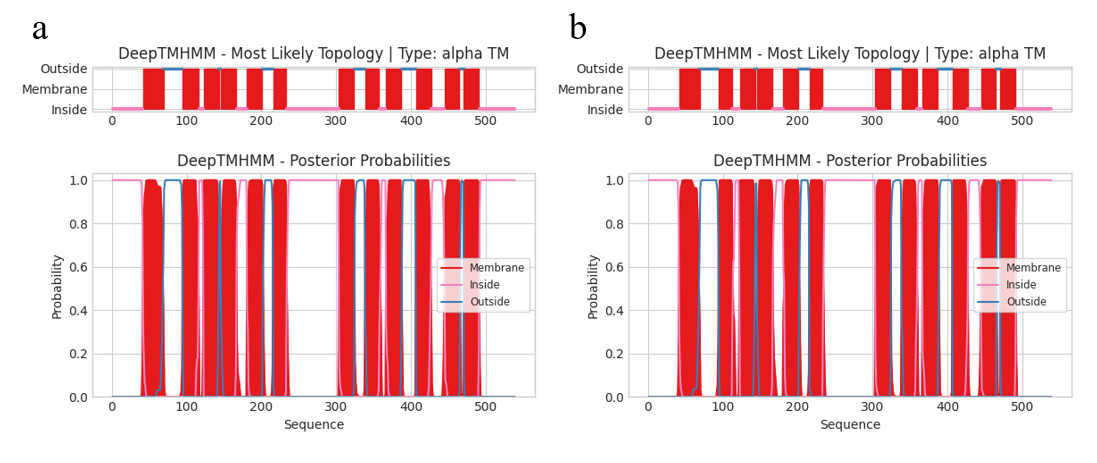
Fig. S5 Transmembrane structure prediction of Xt3 and Xt7 (a: transmembrane structure prediction of Xt3; b: Transmembrane structure prediction of Xt7)
